# A Homogenous Bioluminescent System for Measuring GTPase, GTPase Activating Protein, and Guanine Nucleotide Exchange Factor Activities

**DOI:** 10.1089/adt.2015.643

**Published:** 2015-10-01

**Authors:** Subhanjan Mondal, Kevin Hsiao, Said A. Goueli

**Affiliations:** ^1^ Research and Development, Promega Corporation, Madison, Wisconsin.; ^2^ Department of Pathology and Laboratory Medicine, University of Wisconsin School of Medicine and Public Health, Madison, Wisconsin.

## Abstract

GTPases play a major role in various cellular functions such as cell signaling, cell proliferation, cell differentiation, cytoskeleton modulation, and cell motility. Deregulation or mutation of these proteins has considerable consequences resulting in multiple pathological conditions. Targeting GTPases and its regulators has been challenging due to paucity of convenient assays. In this study, we describe a homogenous bioluminescent assay for monitoring the activities of GTPase and its immediate regulators: GTPase activating proteins (GAPs) and guanine nucleotide exchange factors (GEFs). Since Mg^2+^ plays a critical role in influencing the affinity of GTPases with guanosine triphosphate/guanosine diphosphate (GTP/GDP) and the process of nucleotide exchange, manipulating Mg^2+^ concentrations in the GTPase reaction buffer allows continuous progression of the GTPase cycle and faster hydrolysis of GTP. The assay relies on enzymatic conversion of GTP that remains after the GTPase reaction to ATP and detection of the generated ATP using the luciferin/luciferase combination. The GTPase/GAP/GEF-Glo assay system enables monitoring of GTPase, GAP-stimulated GTPase, GAP, and GEF activities. The system can also be used to analyze these proteins when expressed in cells as fusion proteins by performing the assay in a pulldown format. The assays showed minimal false hits upon testing for compound interference using the library of pharmacologically active compounds and its robustness was demonstrated by a high Z′-factor of 0.93 and CV of 2.2%. The assay system has a high dynamic range, formatted in a convenient add–mix–read, and applicable to high-throughput screening.

## Introduction

Small GTPases are typically 20–25 kDa in size that shuttle between an active guanosine triphosphate (GTP)-bound and inactive guanosine diphosphate (GDP)-bound conformations. The founding member of the small GTPase super family is the Ras GTPase, which is mutated in about 15% of all human tumors. The Ras superfamily GTPases comprise 154 members divided into five subfamilies: Ras, Rho, Rab, Arf, and Ran, and they control diverse cellular functions. The Ras family GTPases mediate signals emanating from cell surface receptors and culminating in transcription, cellular differentiation, and proliferation. The Rho family GTPases regulate cell shape and cytoskeletal processes like cell division and cell migration. Rab and Arf GTPases regulate vesicle-associated processes like vesicle formation, transport, and exocytosis. Ran GTPases regulate nuclear import and export, formation of nuclear envelope, and control of cell division.^1–3^

Structurally, all GTPases share a similar three-dimensional structure called the G-domain responsible for nucleotide binding (GTP or GDP) and GTP hydrolysis. GTPases have very high affinity for both GTP and GDP with a K_d_ in the picomolar to nanomolar range.^4,5^ As a result, cellular GTPases are always present in a nucleotide-bound form and rarely in a nucleotide-free state. Only the active GTP-bound form of GTPases interacts with downstream effector proteins culminating in modulation of cellular signaling. The lifetime of the GTP-bound conformation can be viewed as a timer that determines activation of cellular signaling processes. Thus, GTPases act as an ideal molecular switch between the GTP-bound ON state and GDP-bound OFF state.^6,7^

**Fig. 1.**
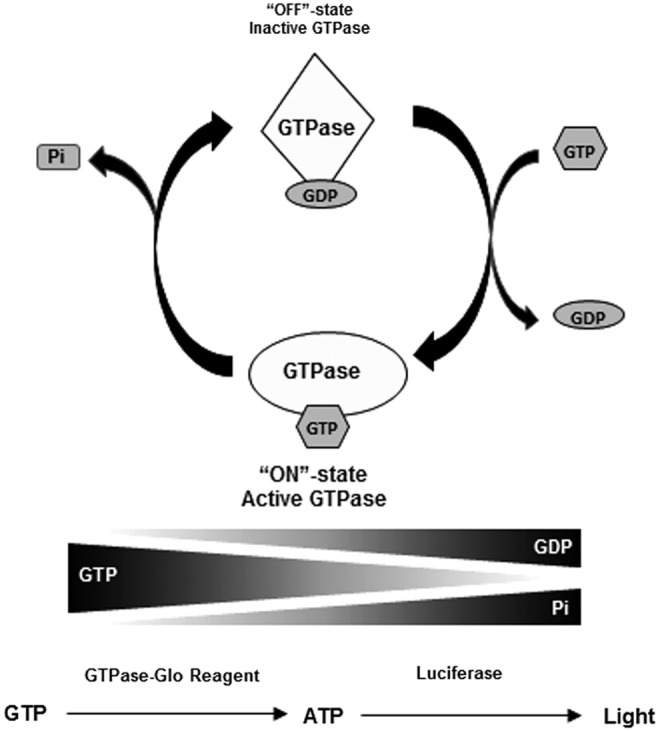
Assay principle for the GTPase-Glo assay. The amount of guanosine triphosphate (GTP) left after the GTPase reaction is used as a readout for the assay. Using an enzyme-coupled reaction, the GTP remaining after the completion of the reaction is converted to ATP and is detected using luciferase/luciferin reaction.

In the cell, the ON–OFF cycle is regulated by two other classes of proteins, guanine nucleotide exchange factors (GEFs) and GTPase activating proteins (GAPs).^8,9^ In a resting cell, the GTPases are in their inactive GDP-bound form. Upon cell stimulation, GEFs activate GTPases by ejecting the GDP out of the active site, creating a transient nucleotide-free state. As the cellular concentration of GTP is 10-fold higher than GDP, GTP immediately occupies the free nucleotide-binding pocket in the GTPase resulting in activation of the GTPase. This process happens in a fraction of a second. GTPases are very slow acting enzymes and would hydrolyze GTP very slowly. The process of GTP hydrolysis is accelerated by GAPs, where GTP is hydrolyzed to GDP and inorganic phosphate (Pi). The GDP remains bound to the GTPase and is converted back to its inactive OFF state with a concomitant release of Pi. This is known as the GTPase cycle (*Fig. 1*).

A primary factor for the lag in drug discovery efforts targeting GTPases and their regulators has been the lack of convenient assays. In this study, we have developed a bioluminescent assay for measuring the GTPase activity, GAP-assisted GTPase activity, GAP activity, and GEF activity. The assay does not require special processing to make nucleotide-free GTPase or GTPase loaded with fluorescent GTP. The system is ideal for the *in vitro* biochemical assay using purified proteins, with optimized reaction buffers for the GTPase, GAP, and GEF activity. The assay uses GTP as the substrate circumventing the use of synthetic fluorescently labeled GTP, which introduces kinetic artifacts.^10^ The assay is simple, formatted in a convenient add–mix–read format with a high dynamic range, and is ideal for high-throughput screening.

## Materials and Methods

### Recombinant Proteins and Other Reagents

Recombinant proteins NF1-333 (containing residues 1,198–1,530 of NF1 protein), RhoA, Rab5A, Ran, RCC1, and RapGAP (Rna1p, GAP for yeast ortholog of mammalian Ran GTPase) were obtained from Jena Biosciences GmbH (Jena, Germany), the Ras protein was obtained from Millipore (Billerica, MA), the Rheb protein and guanosine-S′ [(β,γ)-methylano] triphosphate (GMP-PCP) were obtained from Sigma-Aldrich (St. Louis, MO).

### Expression of Nucleoside Diphosphate Kinase

Saccharomyces cerevisiae nucleoside diphosphate kinase (NDPK, EC 2.7.4.6) was cloned in the *Escherichia coli* expression vector pFN6K encoding an N-terminal MKHQHQHQAIA (HQ-) tag. Protein was expressed in KRX *E.coli* cells and purified using an immobilized metal affinity chromatography HisLink™ Resin (Promega, Madison, WI). Purified protein was stored in 20 mM HEPES (pH 7.5), 50 mM NaCl, 2 mM MgCl_2_, 1 mM DTT, and 50% glycerol.

### Reaction Buffers

The GTPase/GAP reaction buffer is designed for performing the GTPase and GAP-mediated GTPase activity assays. The assay is based on continuous progression of the GTP cycle and GTP hydrolysis. Since we do not use GEF proteins for the progression of the GTPase cycle, when we do the GTPase and GAP-mediated GTPase reaction we use ethylenediaminetetraacetic acid (EDTA) to chemically mimic the role of GEFs to chelate Mg^2+^ from the active site of the GTPase leading to nucleotide release. This buffer would then allow continuous progression of the GTPase cycle and GTP hydrolysis. The composition of the GTPase/GAP reaction buffer is 50 mM Tris-HCl, pH 7.5, 50 mM NaCl, 20 mM EDTA, and 5 mM MgCl_2_.

For GEF reactions, the nucleotide exchange is done in the presence of GEF proteins and does not require a low-Mg^2+^ buffer. The composition of the GEF reaction buffer is 50 mM Tris-HCl, pH 7.5, 50 mM NaCl, 1 mM EDTA, and 10 mM MgCl_2_.

### *In Vitro* GTPase Assays

Upon completion of the GTPase reaction, either in the GTPase/GAP reaction buffer or the GEF reaction buffer, equal volume of GTPase-Glo reagent is added. The GTPase-Glo reagent contains an enzyme that converts the remaining GTP after the GTPase reaction to ATP. Subsequently, the ATP generated is detected by the luciferase/luciferin-based reagent.

### Measurement of NF1 GAP Activity from Cells Expressing Recombinant NF1

NF1-333 (RasGAP-domain) and GST (control) were expressed as a HaloTag™ fusion in bacteria. Induced cells were pelleted and resuspended in a HaloTag Purification buffer containing 50 mM HEPES, pH7.5, and 150 mM NaCl. Lysozyme and RQ1 DNase were added and incubated on ice for 20 min. Cells were lysed by sonication and 2 mM ATP/10 mM MgCl_2_ was added to the sonicated cell lysates and incubated at 37°C for 10 min to get rid of chaperons that may associate with the expressed proteins. Cell lysates were centrifuged at 10,000 *g* for 15 min and supernatant was collected in a fresh tube. Washed HaloLink Resin was added to the cell lysate and incubated at room temperature for 1 h. Upon binding of the expressed proteins with the beads, the HaloLink Resin was washed several times in the HaloTag Purification Buffer; this also removes the ATP that was added to remove chaperones. The NF1- or GST-coated beads were then incubated in the GTPase/GAP reaction buffer containing 10 μM GTP, wild-type Ras, Ras^G12V^, no Ras, or Rheb and incubated for 1 h at room temp. After the GAP/GTPase reaction, the beads were centrifuged and the supernatant was dispensed in a 96-well plate in duplicate. To this equal volume of GTPase-Glo reagent was added and incubated for 30 min. Then, twice as much the volume of detection reagent was added and luminescence was recorded using the GloMax Discover plate reader after 10 min of incubation.

### Compound Library Screening

The Library of Pharmacologically Active Compounds (LOPAC) chemical library (Cat. No. LO4100; Sigma-Aldrich) containing 1,280 compounds was screened in quadruplicate at a final compound concentration of 10 μM in the presence of 5 μM GTP in the GTPase/GAP reaction buffer. GTP was detected using the GTPase-Glo reagent and luciferase/luciferin detection reagent, as described above. The effect of chemical inhibitors on the activity of NDPK and subsequent luciferase/luciferin-mediated ATP detection was analyzed.

### Data Analysis

Z-factors for individual assays were calculated as 1–3 (σ_p_+σ_n_)/(μ_p_-μ_n_), where *σ* is the standard deviation, *μ* is the mean, *p* is positive control, and *n* is negative control. Z′ factors greater than 0.50 are indicative of a highly robust assay.^11^ The coefficient of variation (% CV) was calculated as (σ/μ)×100.

## Results

### Assay Principle

As a consequence of the GTPase cycle, GTP hydrolysis leads to reduction in the amounts of GTP and formation of GDP and Pi. The GDP remains bound to the GTPase, and the Pi is released. Mg^2+^ plays a critical role in influencing the high-affinity binding of guanine nucleotides with GTPases. Chelation of Mg^2+^ using EDTA drives the GTPase cycle without the requirement for GEF proteins. Thus, using a reaction buffer that has an effective low Mg^2+^ concentration, GTPase and GAP-assisted GTPase can be performed without the need for GEF proteins. GEF interacts with GTPase and sterically occludes the Mg^2+^ out of the active site facilitating the GTPase cycle. To monitor the GEF activity, EDTA is not required in the reaction buffer; the GTPase cycle required GTPase and GAP in addition to the GEF proteins.^12–15^

For analysis of the GTPase activity, one could measure the decrease in GTP levels or increase in Pi or bound GDP. Some currently used methods for analysis of GTPase activity rely on chromogenic detection of the Pi using Malachite Green.^16^ These assays have poor sensitivity, and Pi is a common contaminant in various buffers that cause high backgrounds. The GTPase/GAP/GEF-Glo system allows the measurement of remaining GTP after the hydrolysis of GTP in a GTPase reaction. After the completion of the reaction, the remaining GTP is converted to ATP by an enzyme-coupled reaction using NDPK and ADP.^17–20^ The ATP formed is then detected using a thermostable luciferase (Ultra-Glo^®^ Recombinant Luciferase). A high GTPase activity would lead to more GTP hydrolysis and less GTP remaining after the GTPase reaction leading to a lower ATP production and light output. On the other hand, lower GTPase activity would lead to a lower GTP hydrolysis and a larger portion of the GTP remain, which would be converted to ATP by the GTPase-Glo reagent and would generate more light output (*Fig. 1*).

**Fig. 2.**
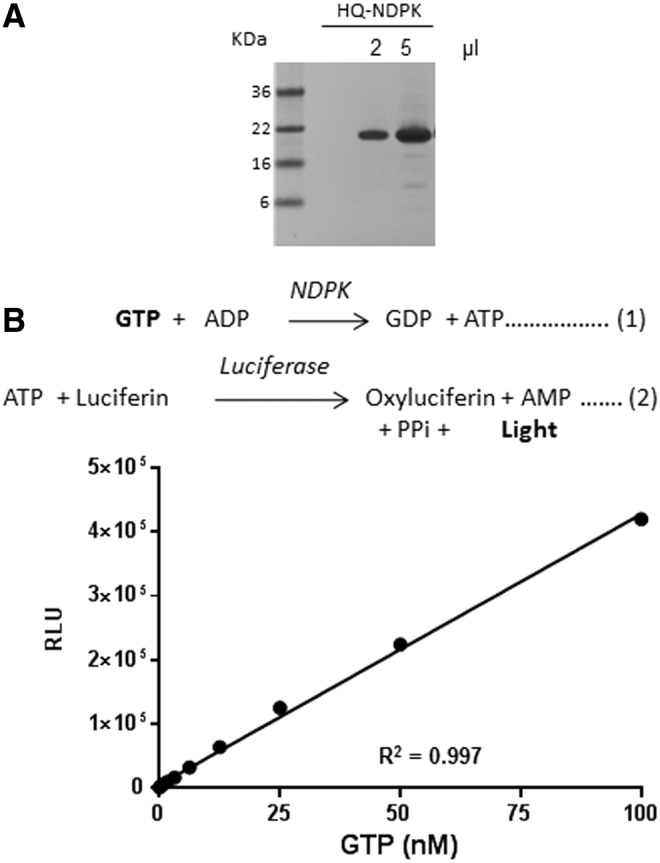
Use of nucleoside diphosphate kinase (NDPK) in detection of GTP. **(A)** Expression and purification of His-Tagged NDPK (from *Saccharomyces cerevisiae*) in KRX *Escherichia coli* cells. Two and five microliters of the purified protein along with a molecular weight marker was resolved by SDS PAGE and stained with Coomassie Brilliant Blue. **(B)** Principle for the use of NDPK in a coupled bioluminescent reaction to detect GTP (*top*). GTP is titrated in water. To it, equal volume of GTPase-Glo reagent containing 1 μg/mL NDPK and 5 mM ADP in GTPase-Glo buffer was added. The reaction was allowed for 30 min. ATP that is generated in the reaction is detected using a luciferin/luciferase-based ATP detection reagent and luminescence is recorded within 10 min (*bottom*). Data represent mean±standard error (SE) (*n*=3).

The GTPase reaction is initiated by addition of GTP in the appropriate reaction buffer containing GTPase in the presence or absence of GAP and GEF. This will lead to consumption of the GTP. Upon completion of the GTPase reaction, an equal volume of GTPase-Glo reagent is added. The GTPase-Glo reagent contains NDPK and ADPs that convert the remaining GTP to ATP and GDP. The ATP formed, inversely correlates with the GTPase activity and is detected using the detection reagent containing a luciferase/luciferin mix (*Fig. 2*). We expressed the NDPK from *S.cerevisiae* and tested its activity by titrating GTP in the two reaction buffers using the GTPase-Glo reagent. The assay was able to detect ∼10 nM GTP (∼50 pico mole GTP) with a signal: noise ratio >3 (*Fig. 2*).

**Fig. 3.**
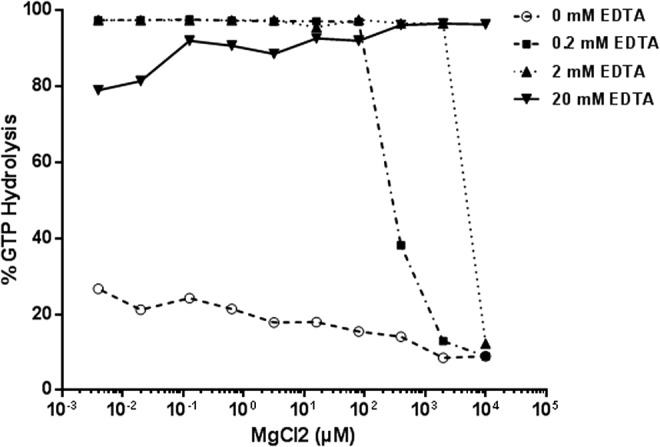
Optimization of Mg^2+^ and ethylenediaminetetraacetic acid (EDTA) in GTPase/GAP reaction buffer. MgCl_2_ was titrated in buffer containing 50 mM NaCl and 50 mM Tris-HCl, pH 7.5. To it, different amounts of EDTA were added to a final concentration of 0, 0.2, 2, or 20 mM. GTPase/GAP reaction was prepared in the different reaction buffer containing 1 μM Ras, 0.5 μM NF1, and 5 μM GTP. GAP-stimulated GTPase reaction was allowed for 2 h. GTPase activity was measured after the addition of GTPase-Glo reagent and detection reagent. % GTP hydrolysis in buffer containing 20 mM EDTA and 5 mM MgCl_2_ was considered as 100%. Data represent mean of experiment in duplicate. GAPs, GTPase activating proteins.

Nucleotide exchange can also be chemically induced using a divalent metal ion chelator-like EDTA that could chelate the Mg^2+^ at the active site leading to nucleotide exchange. To optimize the Mg^2+/^EDTA concentration in the reaction buffer used in the GTPase/GAP reaction, we titrated Mg^2+^ from 0 to 10 mM concentration in the buffer containing 50 mM Tris HCl, pH 7.5, and 50 mM NaCl with 0, 0.2, 2, or 20 mM EDTA. GAP-stimulated GTPase reaction containing 1 μM Ras and 0.5 μM NF1-333 in 5 μM GTP was performed in a reaction buffer containing MgCl_2_ and EDTA. As expected, in solutions containing no EDTA there is little or no GTP hydrolysis as there is no nucleotide exchange. Presence of EDTA in buffers resulted in greater GTP hydrolysis. A concentration of 20 mM EDTA and 5 mM MgCl_2_ was chosen for the GTPase/GAP reaction buffer (*Fig. 3*). The GEF reaction buffer contains 1 mM EDTA and 10 mM MgCl_2_, the high net Mg^2+^ concentration allows nucleotide exchange in the presence of GEF proteins.

### Measurement of GTPase Activity

**Fig. 4.**
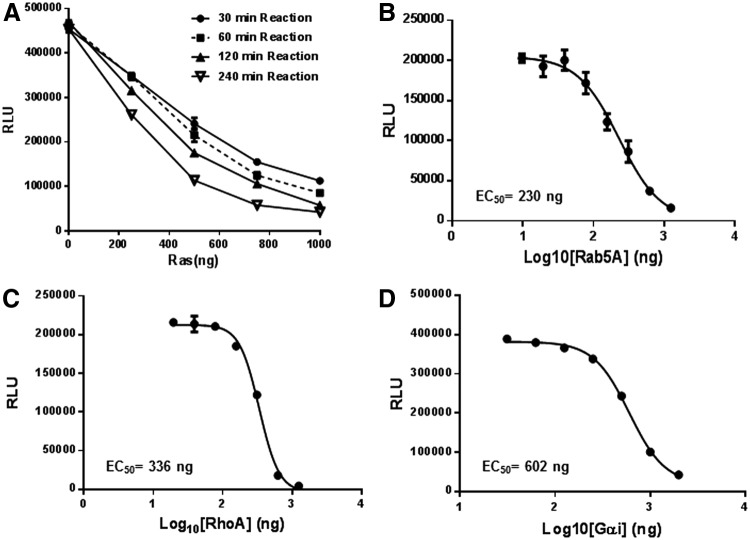
Measurement of intrinsic GTPase activity. **(A)** Intrinsic GTPase activity of Ras at different concentrations for different times. Intrinsic GTPase activities at different concentrations of **(B)** Rab5A, **(C)** RhoA, and **(D)** G_αi_ using a 1-h incubation time using methods described in Materials and Methods. Data represent mean±SE (*n*=3).

Intrinsic GTPase activity of GTPases is extremely slow. To monitor their activity, GTPases (wild-type Ras, RhoA, Rab5A, and G_α_i) were serially diluted in the GTPase/GAP reaction buffer and 2.5 μL of the diluted enzymes were dispensed into wells of a 384-well plate. To this, 2.5 μL of GTP solution containing 10 μM GTP in the GTPase/GAP reaction buffer is added to initiate the GTPase reaction in a total reaction volume of 5 μL. To study the appropriate incubation time for GTPase reactions, we used Ras as a representative example. The Ras GTPase reaction is incubated at room temperature for the indicated time (30, 60, 120, and 240 min). To the completed GTPase reaction, 5 μL GTPase-Glo reagent was added and incubated for 30 min. Thereafter, 10 μL detection reagent was added, and after 5–10 min of incubation, luminescence was recorded using the GloMax Discover multimode plate reader (*Fig. 4A*). As expected, a longer incubation time for the GTPase allows higher GTP hydrolysis. Hereafter, for most experiments we used an incubation time for the GTPase reaction between 1 and 2 h. Using an incubation time of 1 h, we then tested the intrinsic GTPase activity of small GTPase Rab5A (*Fig. 4B*) and RhoA (*Fig. 4C*), as well as the heterotrimeric GTPase G_α_i (*Fig. 4D*). In all cases, we observed increased GTP hydrolysis with an increasing GTPase concentration.

### Measurement of GAP-Stimulated GTPase Activity

GTPase activating proteins (GAPs) accelerate the GTP hydrolysis mediated by GTPases by several orders of magnitude. To test the activity of GAP-stimulated GTPase activities, we used NF1-333 and RanGAP with their cognate GTPase Ras and Ran, respectively. NF1 is a multidomain protein that acts as a GAP for Ras. Inactivating mutations in the NF1 gene leads to neurofibromatosis type 1, a type of tumor of the nervous system.^21–24^

**Fig. 5.**
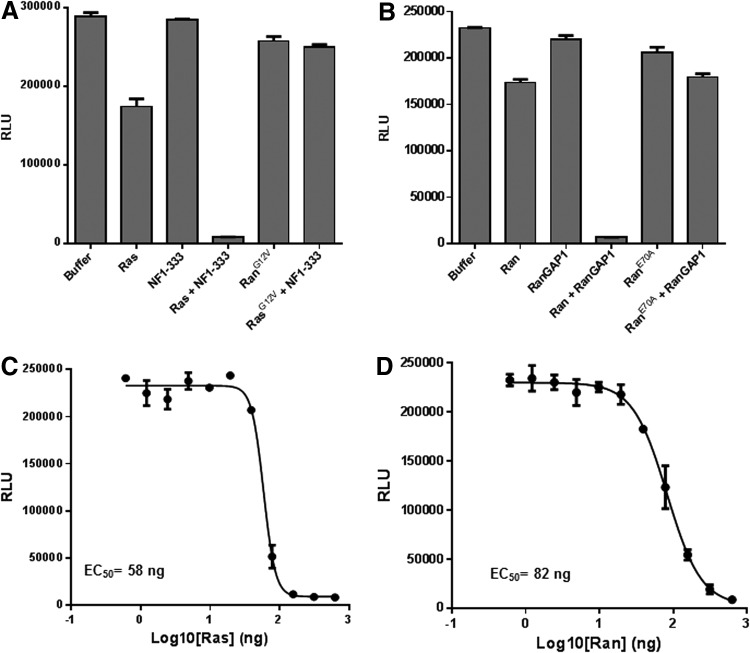
Measurement of GAP-stimulated GTPase activity. **(A)** Effect of NF1-333 on the GTPase activity of wild-type Ras and ^G12V^Ras. **(B)** Effect of RanGap on the GTPase activity wild-type Ran and ^E70A^Ras. **(C)** GTPase activity of different concentrations of wild-type Ras stimulated by 0.5 μM NF1-333. **(D)** GTPase activity of different concentrations of wild-type Ran stimulated by 0.5 μM RanGAP1. Assays are performed using methods described in Materials and Methods. Data represent mean±SE (*n*=3).

Ras^G12V^ and Ran^E70A^ mutants are constitutively active forms of the GTPases. They are insensitive to GAPs and cannot hydrolyze the bound GTP.^25–28^ Reaction containing 2 μM GTPase (wild-type or mutant Ras/Ran), 0.5 μM GAP (NF1-333 or RanGAP), 5 μM GTP, and 1 mM DTT in the GTPase/GAP reaction buffer was set in a final reaction volume of 10 μL. The reaction was incubated for 90 min at room temperature, and the remaining GTP was detected using methods described above (*Fig. 5A, B*). The relative light units (RLU) reading for the buffer control, containing no proteins, represent the total amount of input GTP. We observed that there was a small amount of GTP hydrolyzed by Ras and Ran alone representing their intrinsic GTPase activity. The GAPs, NF1 and RanGAP, do not possess any intrinsic GTPase activity, but when present with their cognate GTPases, there was significant GTP hydrolysis. It is also important to note that constitutively active GTPases Ras^G12V^ and Ran^E70A^ do not show GAP-stimulated GTPase activities (*Fig. 4A*, *B*). One mechanism to target oncogenic Ras is to identify chemical probes that facilitate GTP hydrolysis by mutant Ras in the presence of GAP. The GTPase-Glo assay allows identification of such chemical probes in a high-throughput format.

Next, GTPases (Ras and Ran) were titrated in the presence of a fixed concentration of their cognate GAPs. For this, GTPase (either wild-type Ras or wild-type Ran) was serial diluted in the GTPase/GAP reaction buffer, and 5 μL aliquots were dispensed into wells of a 384-well plate. To this, 5 μL of GAP-GTP solution containing 0.2 μM NF1 (for Ras) or 0.2 μM RanGAP (for Ran) and 10 μM GTP in the GTPase/GAP reaction buffer containing 1 mM DTT was added to each well with a total reaction volume of 10 μL. The GTPase reaction was incubated for 2 h. Upon completion of the reaction, the remaining GTP is detected using the GTPase-Glo reagent and detection reagent. Results show that increasing GTPase concentration, with a constant amount of GAP, increases the GTP consumption (*Fig. 5C, D*).

### Measurement of GAP Activity

**Fig. 6.**
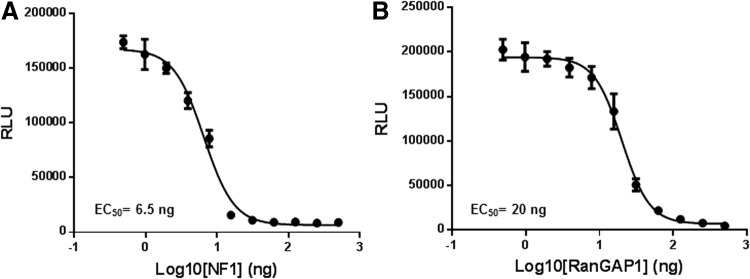
Measurement of GAP activity. **(A)** GAP activity of different concentrations of NF1-333 in reactions containing 0.5 μM wild-type Ras and 5 μM GTP. **(B)** GAP activity of different concentrations of RanGAP1 in reactions containing 1 μM wild-type Ran and 5 μM GTP. Assays are performed using methods described in Materials and Methods. Data represent mean±SE (*n*=3).

To test GAP activities, we titrated GAPs (NF1-333 and RanGAP) in the presence of a fixed concentration of its cognate GTPase. NF1-333 or RanGAP1 were serially diluted in the GTPase/GAP reaction buffer. To 5 μL of GAP, 5 μL of GTPase-GTP solution containing 1 μM GTPase (Ras/Ran), 10 μM GTP, and 1 mM DTT in the reaction buffer was added and incubated for 2 h. To the completed reaction, 10 μL GTPase-Glo reagent was added and incubated for an additional 30 min, and luminescence was recorded after addition of 20 μL of detection reagent. Results indicate that increasing GAP concentration with a constant amount of GTPases causes more GTP consumption resulting in a lower light output (*Fig. 6A*, *B*), thus demonstrating activation of GTPase activity by increasing the amount of GAP in the reaction. Z′ Factor is a statistical function commonly used to judge high-throughput screening assay robustness. The formula for Z′ factor takes into consideration standard deviation as well as the difference in the means of the high and low values of an assay. Z′ factors greater than 0.50 are indicative of a robust assay.^11^ For 1 μM Ras and NF1 concentration from 4 to 500 nM, the Z′ was higher than 0.77 and coefficient of variation was between 5% and 15%.

### Competitive Inhibition of GTPase Activity by a Nonhydrolyzable GTP Analog

**Fig. 7.**
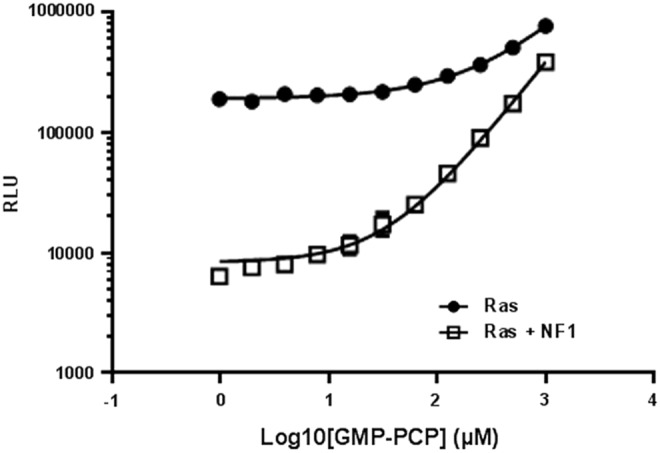
Inhibition of intrinsic GTPase activity and GAP-stimulated GTPase activity by a nonhydrolyzable GTP analog guanosine-S′-[(β,γ)-methylano] triphosphate (GMP-PCP). GMP-PCP was titrated in Ras GTPase reaction (*dark circles*) or NF1-stimulated Ras reaction (*open squares*). After the GTP-hydrolysis reaction for 1 h, detection of the GTP that remained was detected by methods described above. Data represent mean±SE (*n*=2).

We tested the inhibition of GTPase activity by a nonhydrolyzable GTP analog (GMP-PCP). GMP-PCP was titrated in Ras GTPase or NF1-stimulated Ras reactions. The final reaction contained 1 μM Ras, 0.5 μM NF1, and 5 μM GTP in the GTPase/GAP reaction buffer in a 10 μL reaction. After the GTP-hydrolysis reaction for 1 h, detection of the GTP that remained was detected by methods described above. We observe that by increasing the concentration of GMP-PCP, the GTP-hydrolysis was inhibited resulting in a higher light output (*Fig. 7*). Although GMP-PCP and other competitive GTP analogs may inhibit the GTPase activity at high molar concentrations, such compounds may not be an ideal drug candidate for targeting GTPases as GTPases have a very high affinity for GTP and the cellular concentration of GTP is in the millimolar range.

### Measurement of GAP Activities from Cell Lysates Expressing Recombinant GAP

**Fig. 8.**
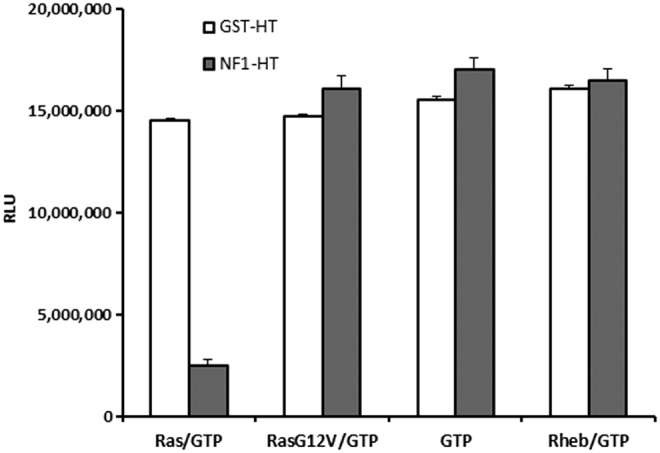
Measurement of GAP activity from cell lysates expressing recombinant GAP. Glutathione-Sepharose beads were coated with GST-HaloTag (control) or GST-NF1-333 and incubated with wild-type Ras, ^G12V^Ras, Rheb, or no GTPase and GTPase reaction initiated by addition of GTP as described in Materials and Methods. GTP hydrolysis was only observed in reactions where NF1-333-coated beads were incubated with wild-type Ras. Data represent mean±SE (*n*=2).

In this study, we present a method where GTPase, GAP, or GEF can be analyzed from cell extracts using pulldown of tagged proteins and performing the GTPase-Glo assay on proteins bound to beads. NF1-333 and GST (control) were expressed as HaloTag fusion proteins in bacteria and affinity was purified using HaloLink beads.^29,30^ The beads were used to perform GTPase reaction and were detected using the GTPase-Glo system. We observe that only in the presence of wild-type Ras and NF1, a continuous GTP hydrolysis is possible as evident from the drop in light output. The Ras^G12V^ mutant was not able to keep the GTPase cycle running and no GTP was hydrolyzed. It is also important to note that NF1 does not act as a GAP for the Rheb GTPase (the known GAP for Rheb is TSC2), indicating the selectivity of GTPases for their cognate GAP. This also indicates that the assay can not only be used to assay the GTPase activity in cell lysates but also access the specificity of GAPs and GEFs to their cognate GTPases (*Fig. 8*). This approach can be used to analyze GTPase/GAP of GEF activities of uncharacterized proteins and the enzyme activity can be investigated.

### Measurement of GEF Activity

GEFs mediate the exchange of GDP bound to the inactive GTPases with GTP, thereby making them active. To test for GEF activity, we used RCC1, the GEF for Ran GTPase as a representative.^31–33^ The reaction buffer for GEF activities is different from the one used for analyzing intrinsic GTPase and GAP activities. The GEF reaction buffer contains higher amounts of free Mg^2+^, and the nucleotide loading is catalyzed solely by the GEF.

**Fig. 9.**
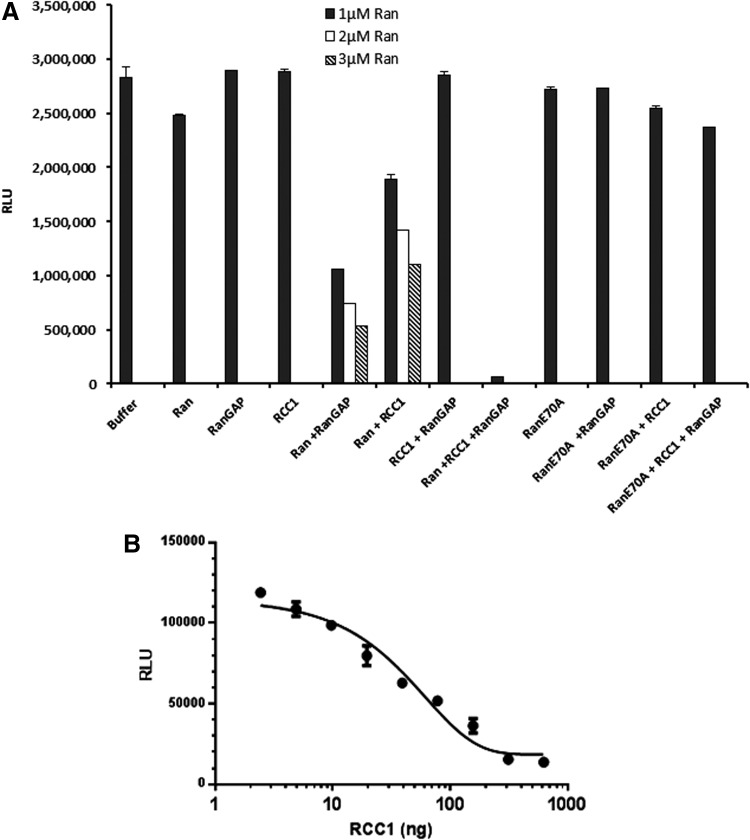
Measurement of GEF activity. **(A)** Effect of RanGEF RCC1 on the GTPase activity of wild-type Ran and ^E70A^Ras in the presence or absence of RanGAP. **(B)** GEF activity of different concentrations of RCC1 in reactions containing 0.5 μM wild-type Ran, 0.25 μM RanGAP, and 5 μM GTP. Assays are performed using methods described in Materials and Methods. Data represent mean±SE (*n*=3). GEF, guanine nucleotide exchange factors.

To analyze the GEF activity, a reaction containing 1 μM Ran or Ran^E70A^, 0.5 μM RanGAP, 0.5 μM RCC1, 5 μM GTP, and 1 mM DTT in the GEF reaction buffer was set. Various combinations of GTPase, GEF, and GAPs were used. The final reaction volume was 10 μL. The reaction was incubated for 90 min at room temperature, and GTP remaining after the completion of the reaction was determined using methods described above. The RLU reading for the buffer control represents the total amount of input GTP. We observed that there was a small amount of GTP hydrolyzed by Ran, which represents an intrinsic GTPase activity. RanGAP and RCC1 do not possess any intrinsic GTPase activity, but when included with Ran we observe significant GTP hydrolysis, which is dependent on the concentration of Ran GTPase. In the presence of all components required for effective GTPase cycling, Ran, RanGAP, and RCC1, all the input GTP is hydrolyzed resulting in a very low light output. We also observed that in the presence of Ran and RCC1, there was GTP hydrolysis, which was increased by increasing the amount of the Ran GTPase. It is also important that the constitutively activated form of the GTPases Ran^E70A^ is not able to hydrolyze GTP even in the presence of RCC1 GEF and RanGAP (*Fig. 9A*). This experiment also indicates that for measuring optimum GEF activity using the GTPase-Glo system, a cognate GAP should be included.

Furthermore, we tested the GEF activity of RCC1 by titrating RCC1 in the presence of a fixed concentration of its cognate Ran and RanGAP. RCC1 was serially diluted in the GEF reaction buffer, and the GTPase reaction was performed in solution containing 1 μM Ran, 0.5 μM RanGAP, 10 μM GTP, and 1 mM DTT in the reaction buffer with a total reaction volume of 10 μL. The reaction is incubated for 2 h, and the amount of GTP consumed was determined using the GTPase-Glo reagent as described above. Results indicate that by increasing RCC1 GEF concentration, the GTP consumption is increased resulting in a lower light output (*Fig. 9B*).

### LOPAC Screening Using the GTPase-Glo Assay

The LOPAC library is a collection of 1,280 pharmacologically active compounds from 56 pharmacological classes with well-characterized activities. The library was screened to identify potential chemicals that may inhibit the assay by either inhibiting NDPK or luciferase. The library was screened using 5 μM GTP in the GTPase/GAP reaction buffer containing LOPAC chemicals at a final compound concentration of 10 μM using methods described above. The ATP generated was detected using a luciferase/luciferin-based ATP detection reagent, and luminescence readings were recorded using a luminometer. The assay was performed in low-volume 384-well plates in quadruplet. Since the affinity of GTPases for GDP and GTP is in the picomolar range, it is apparent that a desirable inhibitor should have an affinity similar to or lower than this concentration. Therefore, developing a GTPase assay that uses 5 μM GTP will preferentially detect uncompetitive/noncompetitive inhibitors over competitive inhibitors.

**Fig. 10.**
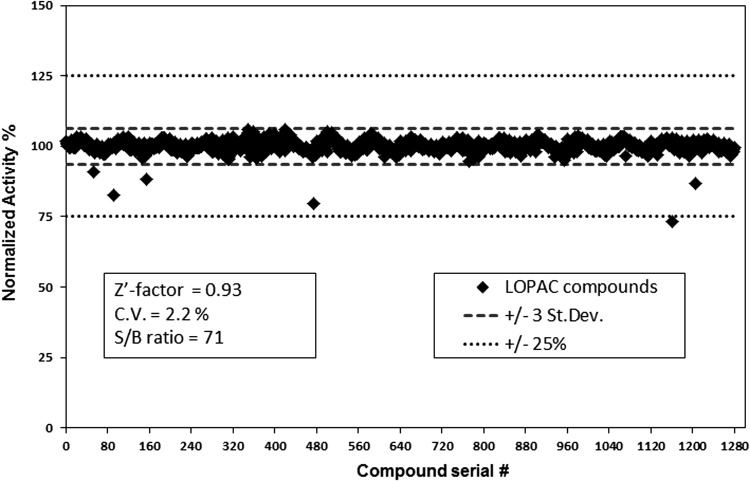
Library of pharmacologically active compounds (LOPAC) screening. The LOPAC chemical library containing 1,280 compounds at a final compound concentration of 10 μM in DMSO was screened in quadruplicate in the presence of 5 μM GTP in GTPase/GAP reaction buffer. GTP was detected using GTPase-Glo reagent and luciferase/luciferin detection reagent as described in Materials and Methods. There were only two compounds, which were known luciferase inhibitors that showed 25% inhibition of the assay validating the assay for high-throughput screening. The robustness of the assay to detect GTP was analyzed by measuring the Z′ factor and % CV. *Z*′ factor using the equation *Z*=1−3×(δ_p_+δ_n_)/(|μ_p_−μ_n_|), where δ_p_ and δ_n_ are the standard deviations of positive and negative controls and μ_p_ and μ_n_ are the means of positive and negative controls. Coefficient of variation was calculated as (σ/μ)×100. Signal:background ratio of 71. The normalized data after background subtraction are represented.

We found that only two compounds, which were known as luciferase inhibitors, interfered with the assay and reduced the luminescent signal by only 20%–25%. None of the compounds in the LOPAC library affected the NDPK activity. We observed a Z′ of 0.93 for the GTPase-Glo assay. Furthermore, the coefficient of variation was 2.2%, indicating little well to well variation (*Fig. 10*). The LOPAC analysis confirms that the assay is not only resistant to a wide variety of pharmacologically active compounds but also highly robust and reproducible, making it ideal for high-throughput screening.

## Discussion

The structural differences between GDP-bound GTPase and GTP-bound GTPase are very subtle and primarily confined to two regions called switch I and switch II. In the GTP-bound form, the switch I and II are less flexible and can interact with downstream effector molecules and transmit signal transduction, but when the GTP is hydrolyzed, the switch I and II are highly flexible, which does not allow proper binding with downstream effector molecules and the signal transduction is shut off. Guanine nucleotides interact with high affinity to GTPases due to a conserved DXXG motif in the G-domain. Interactions between the P-loop and an Mg^2+^ ion in the active site also contribute significantly to the high-affinity interaction of G-domains with guanine nucleotides. When GEFs interact with the switch I and switch II, they kick the Mg^2+^ out of the active site making the interaction of guanine nucleotide with GTPase weaker, allowing nucleotide exchange by cellular GTP, which is abundantly present at millimolar concentrations leading to activation of the GTPase.^34^

For GTP hydrolysis, a water molecule is positioned optimally in the active site for nucleophilic attack to the γ-phosphate of the GTP molecule. GAPs stabilize the Glu61 of Ras, which is critical for the nucleophilic attack. In addition, GAPs provide a critical Arg residue in the active site (also called Arg-finger) that stabilizes the transition state intermediate by neutralizing the charge on the γ-phosphate.^35^ Mutations in the Glu61 of Ras frequently occur in tumors that abolish the GAP-mediated GTP hydrolysis. Mutations on Glycine residues at position 12 and 13 of Ras also lead to oncogenic forms. These mutations cause a steric block for proper orientation of the Arg-finger in the active site also leading to inhibition of GAP-assisted GTP hydrolysis. Upon inhibition of GTP hydrolysis by these GTPases mutants (constitutively activated GTPases), they remain bound to GTP and can transmit signal to downstream effector proteins even without any physiological stimulus. Thus, when Ras activation is uncontrolled by such constitutively activated mutations, there is uncontrolled cell proliferation leading to cancer.

GTPases play a critical role in signal transduction and alteration of their function leads to various clinical consequences. The most serious among these are Ras-driven cancers. There is a very high incidence of Ras mutations in cancer with no effective therapies. Patients with Ras-driven cancers are excluded from treatment with targeted therapies.^36,37^ This is largely due to the lack of available drugs that target oncogenic Ras directly or indirectly. Efficient inhibition of signaling mediated by oncogenic Ras mutants is considered as the Holy Grail in cancer therapy. Drug discovery efforts to directly target oncogenic Ras suffered a setback after the failure of farnesyltransferase inhibitors to meet clinical safety and efficacy.^38^ Recently, the area has gained substantial momentum, and novel methods of targeting oncogenic Ras are being actively investigated.^39–43^

In addition to oncology, GTPases are implicated in a variety of other pathologies. An example of the clinical importance of GTPases is observed in host–pathogen interactions. Microbes during coevolution with host have found that affecting the host GTPase is one of the best ways to hijack host cellular machinery critical for pathogenesis. Several virulence factors are known to affect host GTPase by causing post-translational modifications on GTPases.^44–46^

One factor that has hindered advancement in drug discovery efforts in GTPase research is the lack of simple, robust assay systems. Technical options for analysis of GTPases have been put forward, but have not seen much progress despite the unmet medical needs in oncology, infection, immunity, diabetes, and other pathologies. Although GTPases and their regulators GEFs and GAPs are relevant therapeutic drug targets, effective chemical probes that modulate their activities have not been identified. This is primarily due to lack of convenient assays. *In vitro* GTPase assays using recombinant proteins require GTPase either in a nucleotide-free state or loaded with radiolabeled or fluorescently labeled GTP.^47,48^ The GEF activity has been measured by incorporation or displacement of a fluorescently labeled GTP into a GTPase. GTPase or GAP activity is generally measured by the hydrolysis of [P^32^]-γ-phosphate-labeled GTP that releases the terminal radioactive Pi.^49,50^ Although radioactive assays are sensitive, they are expensive and hazardous, requiring highly trained personnel. Assays using fluorescent assays are safe, but require high concentrations of protein and offer a narrow dynamic range.^4,51–53^ Recent evidence suggests that fluorescent GTP analogs like Mant-GDP [where Mant is 2′(3′)-O-(N'methylanthraniloyl)] and Mant-GTP may alter the hydrolytic properties of GTPases as well as the interaction of GTPases with their cognate GEFs and GAPs.^10,54,55^ Monitoring the activity of RGS proteins, GAP modulators of G-protein-coupled receptors have been described using anti-GDP antibodies and a GDP-conjugated fluorescent tracer. The method relies on the binding competition between GDP that is released from the GAP activity of RGS and the GDP tracer to the GDP antibody using fluorescent polarization measurements. Critical factors for assay performance include extreme differentiation between binding of the antibody to GDP vs. GTP, since the latter is the substrate for the reaction, and the ratio of antibody to GTP concentrations needs to be optimized due to some cross reactivity of the antibody to GTP.^56^ This and other methods that are based on different detection systems can be used as orthogonal methods for verification of the hits obtained by our method.^53,56^

Several cell-based assays have also been designed using effector-binding domains that bind to the GTP-bound form of certain GTPase. This assay is based on affinity precipitation using resin coated with the binding domains followed by detection using western blotting. These assays are labor intensive, difficult to perform in replicates, and not amenable to high-throughput screening.

The GTPase-Glo assay system overcomes many of the difficulties encountered in the currently available GTPase assay methods and allows a convenient system to analyze the GTPase activity as well as GAP-stimulated GTPase activity, GAP activity, and GEF activity. The system can also be used to analyze these proteins expressed in cells as a fusion protein performing the assay in a pulldown format. Furthermore, there were minimal false hits when tested for compound interference using the LOPAC, and its robustness was demonstrated as indicated by a high Z′-factor of 0.93 and CV of 2.2%. Since the assay uses multiple enzymes, which might result in a few false hits depending on the composition of the chemical library, we have optimized assay formulations to make it robust to the interference from compounds. These include the use of high concentration of luciferin and an Ultraglo luciferase enzyme that has been proven in many other luminescent assays to be resistant to diverse chemical compounds due to its unique sequence and buffer formulations.^57^ These features make the system highly useful for high-throughput applications to identify therapeutically useful chemicals targeting GTPases and their regulator GEFs and GAPs.

## Abbreviations Used

GAP: GTPase activating protein
GEF: guanine nucleotide exchange factor
EDTA: ethylenediaminetetraacetic acid
LOPAC: library of pharmacologically active compounds
NDPK: nucleoside diphosphate kinase
GTP: guanosine triphosphate
GDP: guanosine diphosphate
GMP-PCP: guanosine-S′ [(β,γ)-methylano] triphosphate
